# Serum Peroxides and Galectin-3 in Chronic Heart Failure: Cross-Sectional Associations with NT-proBNP and Cardiac Structure

**DOI:** 10.3390/biomedicines14102166

**Published:** 2026-09-24

**Authors:** Samuel Ardelean, Diana E. Buzzi, Andrei-Catalin Zavragiu, Alexandra Gogan, Daniel Rus, Elena-Larisa Zimbru, Andrada Ardelean, Dania Hasanein, Ruxandra Maria Christodorescu, Adrian Sturza, Minodora Andor

**Affiliations:** 1 Doctoral School of Medicine, “Victor Babeș” University of Medicine and Pharmacy of Timișoara, Eftimie Murgu Square, No. 2, 300041 Timișoara, Romania; samuel.ardelean@umft.ro (S.A.); diana.mailat@umft.ro (D.E.B.); andrei.zavragiu@umft.ro (A.-C.Z.); alexandra.gogan@umft.ro (A.G.); daniel.rus@umft.ro (D.R.); elena.zimbru@umft.ro (E.-L.Z.); 2 Department of Cardiology, Institute of Cardiovascular Diseases, Str. Gheorghe Adam No. 13A, 300310 Timișoara, Romania; 3 Department V—Internal Medicine 1, “Victor Babeș” University of Medicine and Pharmacy, Eftimie Murgu Square, No. 2, 300041 Timișoara, Romania; christodorescu.ruxandra@umft.ro (R.M.C.); andor.minodora@umft.ro (M.A.); 4 Multidisciplinary Heart Research Center, Faculty of Medicine, “Victor Babeș” University of Medicine and Pharmacy, Eftimie Murgu Square, No. 2, 300041 Timișoara, Romania; 5 Center of Immuno-Physiology and Biotechnologies, Department of Functional Sciences, “Victor Babeș” University of Medicine and Pharmacy, 300041 Timișoara, Romania; 6 Center for Gene and Cellular Therapies in the Treatment of Cancer—OncoGen, Timiș County Emergency Clinical Hospital “Pius Brînzeu”, 300723 Timișoara, Romania; 7 Department of Rheumatology-Physical Medicine and Rehabilitation Clinic-Municipal Emergency Clinical Hospital, 300020 Timișoara, Romania; andrada.ardelean@rezident.umft.ro; 8 Department of Advanced Cardiology and Hemostaseology Research, “Victor Babeș” University of Medicine and Pharmacy, Eftimie Murgu Square, No. 2, 300041 Timișoara, Romania; dania.hasanein@umft.ro; 9 Research Center, Institute of Cardiovascular Diseases Timișoara, Gheorghe Adam St., No. 13A, 300310 Timișoara, Romania; 10 Department of Functional Sciences-Pathophysiology, Center for Translational Research and Systems Medicine, “Victor Babeș” University of Medicine and Pharmacy of Timișoara, Eftimie Murgu Square, No. 2, 300041 Timișoara, Romania

**Keywords:** heart failure, oxidative stress, serum peroxides, galectin-3, NT-proBNP, biomarkers, left ventricular structure, cardiorenal dysfunction

## Abstract

**Background/Objectives**: Oxidative stress and galectin-3-related fibro-inflammatory processes contribute to heart failure pathophysiology, but their concurrent relationships with natriuretic peptides and structural/cardiorenal parameters remain incompletely characterized. We evaluated baseline associations of serum peroxide burden and galectin-3 with N-terminal pro-B-type natriuretic peptide (NT-proBNP) and related phenotypic parameters in chronic heart failure. **Methods**: This cross-sectional analysis included 57 patients from a prospective single-center cohort. Serum peroxides were quantified using the ferrous oxidation–xylenol orange assay and expressed as nmol H_2_O_2_ equivalents/mL. Associations were assessed using Spearman correlation and linear regression with ln-transformed NT-proBNP. Benjamini–Hochberg correction was applied to eight secondary/exploratory correlations. **Results**: Serum peroxide burden correlated with NT-proBNP (ρ = 0.387, *p* = 0.0095; *n* = 44) and remained significantly associated after adjustment for age, sex, and left ventricular ejection fraction (B = 0.1756, *p* = 0.0420; *n* = 43). After additional estimated glomerular filtration rate (eGFR) adjustment, the effect estimate remained similar, although statistical significance was not reached (B = 0.1646, *p* = 0.0525; *n* = 39). Galectin-3 correlated with NT-proBNP, left ventricular end-diastolic volume index (LVEDVi), and serum uric acid; all associations remained significant after false-discovery-rate correction. Serum peroxide burden and galectin-3 were not significantly correlated. In an exploratory joint model (*n* = 29), both biomarkers remained significantly associated with ln(NT-proBNP) (adjusted R^2^ = 0.4294). **Conclusions**: Serum peroxide burden and galectin-3 showed different cross-sectional association patterns with NT-proBNP and selected structural/cardiorenal parameters. Given the limited sample size, missing data, and exploratory nature of the joint model, these findings should be considered hypothesis-generating and do not establish biological independence or incremental clinical value.

## 1. Introduction

Heart failure is a complex and heterogeneous clinical syndrome encompassing diverse biological and phenotypic profiles across the spectrum of left ventricular ejection fraction (LVEF) [1,2]. Natriuretic peptides, particularly N-terminal pro-B-type natriuretic peptide (NT-proBNP), are central to the diagnostic evaluation and risk stratification of patients with heart failure [3,4]. However, NT-proBNP predominantly reflects myocardial wall stress and hemodynamic burden and may therefore not fully capture the multiple biological pathways contributing to heart failure progression, including oxidative stress, inflammation, and myocardial fibrosis [5,6,7].

Oxidative stress results from an imbalance between the generation of reactive oxygen species and endogenous antioxidant defenses and contributes to myocardial dysfunction, endothelial dysfunction, inflammation, and adverse cardiac remodeling [8,9]. Circulating hydroperoxide concentrations provide a marker of systemic oxidative stress and can be quantified using the ferrous oxidation–xylenol orange (FOX) assay [10,11]. Previous human studies have demonstrated increased circulating oxidative-stress markers in chronic heart failure and positive associations between oxidative-stress indices and natriuretic peptide concentrations, suggesting a relationship between redox imbalance and heart failure severity [12,13].

Galectin-3 is a β-galactoside-binding lectin involved in macrophage activation, inflammation, extracellular matrix remodeling, and myocardial fibrosis [14,15]. In patients with chronic heart failure, elevated circulating galectin-3 concentrations have been associated with adverse cardiac remodeling and adverse long-term clinical outcomes [16,17]. Galectin-3 has therefore been proposed as a circulating marker associated with fibro-inflammatory and remodeling-related processes, whereas NT-proBNP primarily reflects hemodynamic stress and serum peroxide concentrations provide an index of circulating hydroperoxide burden. The present cohort was established for prospective evaluation of patients undergoing sodium–glucose cotransporter-2 (SGLT2) inhibitor initiation; however, the current analysis focuses exclusively on the pre-SGLT2-inhibitor baseline phenotype.

Although oxidative stress and galectin-3 have each been investigated in heart failure, studies examining their concurrent cross-sectional associations with NT-proBNP, cardiac structural parameters, renal function, and inflammatory indices within the same cohort remain limited. Evaluating these biomarkers concurrently may help characterize their respective association patterns without presuming biological independence or clinical complementarity.

Therefore, the present study aimed to evaluate the baseline associations of serum peroxide burden and galectin-3 with NT-proBNP and selected structural, renal, and inflammatory parameters in patients with chronic heart failure. The primary hypothesis was that higher serum peroxide concentrations would be associated with higher NT-proBNP concentrations. As an exploratory analysis, we also evaluated serum peroxide burden and galectin-3 separately and simultaneously in an identical complete-case sample to characterize their respective associations with NT-proBNP.

## 2. Materials and Methods

### 2.1. Study Design and Population

This study represents a cross-sectional analysis of baseline data collected within a prospective, single-center, observational cohort study evaluating patients with chronic heart failure before the initiation of sodium–glucose cotransporter-2 inhibitor therapy. Patients were evaluated in a clinically stable condition, operationally defined for the present study as the absence of clinical evidence of acute heart failure decompensation at the time of baseline assessment and blood sampling. Patients who fulfilled the eligibility criteria were invited to participate at the ASCAR Cardiology Clinic of the Timișoara Municipal Emergency Clinical Hospital, Timișoara, Romania, between 1 July 2025 and 1 July 2026. The present analysis included 57 patients who provided written informed consent. The number and characteristics of eligible patients who declined participation were not systematically recorded; therefore, the participation rate could not be calculated. The total number of individuals screened and the specific reasons for non-enrolment were not systematically recorded and therefore could not be reliably reconstructed. Because not all variables were available for every participant, analysis-specific sample sizes varied according to complete-case availability; missing data were not imputed.

Heart failure diagnosis was based on either a previously established and documented diagnosis or a diagnosis established by the treating cardiologist at enrollment. In patients without a previously established and documented diagnosis, heart failure was diagnosed at enrollment based on compatible symptoms and/or clinical signs together with elevated NT-proBNP concentrations, defined as >300 pg/mL in patients in sinus rhythm and >600 pg/mL in patients with atrial fibrillation. For phenotype-based analyses, patients with available LVEF were classified as having heart failure with reduced ejection fraction (HFrEF; LVEF ≤ 40%), heart failure with mildly reduced ejection fraction (HFmrEF; LVEF 41–49%), or heart failure with preserved ejection fraction (HFpEF; LVEF ≥ 50%), in accordance with the 2021 European Society of Cardiology (ESC) Guidelines [18]. In patients with LVEF ≥ 50%, HFpEF classification was based either on a previously established and documented diagnosis of HFpEF or, when the diagnosis was established at enrollment, on compatible symptoms and/or signs of heart failure, NT-proBNP concentrations above the predefined rhythm-specific thresholds, and echocardiographic evidence of left ventricular diastolic dysfunction, defined by a left atrial volume index > 34 mL/m^2^ in sinus rhythm or >40 mL/m^2^ in atrial fibrillation and/or E/e′ ≥ 9.

Concomitant medication use documented at the baseline T0 assessment was recorded from the study database and included beta-blockers, statins, angiotensin-converting enzyme inhibitors, angiotensin receptor blockers, angiotensin receptor–neprilysin inhibitors, mineralocorticoid receptor antagonists, furosemide, calcium-channel blockers, antiplatelet therapy, oral anticoagulants, and ivabradine. Only baseline assessments performed before SGLT2 inhibitor initiation were included in the current analysis. Because all measurements analyzed in the present study were obtained before SGLT2 inhibitor initiation, the subsequently prescribed SGLT2 inhibitor type and dose were not included in this baseline analysis.

No formal a priori sample-size calculation was performed for the present cross-sectional analysis. The parent prospective cohort had a pragmatic recruitment target of approximately 100 participants. The current analysis included all 57 participants enrolled during the predefined baseline recruitment period and was therefore considered exploratory.

### 2.2. Eligibility Criteria

Adult patients were eligible if they had a diagnosis of chronic heart failure, were considered suitable for treatment with dapagliflozin or empagliflozin according to the treating physician, and provided written informed consent for study participation and biological sample collection. This eligibility criterion reflects the design of the parent prospective cohort, which was established to evaluate patients before and after SGLT2 inhibitor initiation. Accordingly, the present baseline analysis represents a selected SGLT2-inhibitor-eligible chronic heart failure cohort rather than an unselected population of patients with chronic heart failure.

Exclusion criteria at screening included previous treatment with an SGLT2 inhibitor, severe valvular heart disease, active malignancy or ongoing oncological treatment, active systemic inflammatory disease, acute infection or sepsis, estimated glomerular filtration rate (eGFR) < 30 mL/min/1.73 m^2^, glycated hemoglobin > 10%, pregnancy, and breastfeeding. These eligibility criteria were prespecified for the parent prospective cohort and were not developed specifically for the present cross-sectional biomarker analysis. The eGFR threshold of <30 mL/min/1.73 m^2^ was therefore a predefined exclusion criterion of the parent study.

### 2.3. Ethical Considerations

The study was conducted in accordance with the Declaration of Helsinki and approved by the Scientific Research Ethics Committee of the “Victor Babeș” University of Medicine and Pharmacy, Timișoara, Romania (approval no. 28, 10 June 2025). Written informed consent was obtained from all participants before enrollment and covered study participation, biological sample collection, research use of clinical and laboratory data, and publication of anonymized results.

### 2.4. Blood Sampling and Serum Processing

Venous blood samples were collected at baseline, before SGLT2 inhibitor initiation. Serum peroxide, galectin-3, and NT-proBNP samples were obtained during the same baseline blood-draw session, using separate collection tubes according to assay-specific requirements. Blood samples were collected in the fasting state.

Samples intended for serum peroxide assessment were collected into red-top tubes without anticoagulant and allowed to clot at room temperature for approximately 15–20 min before centrifugation at 2000 rpm for 20 min. The resulting serum was then separated into a clean tube and analyzed immediately, without prior freezing or storage. No freeze–thaw cycle occurred before peroxide measurement. Samples intended for galectin-3 measurement were collected separately into yellow-top serum collection tubes, kept refrigerated until transport, delivered to an external laboratory within 30 min of collection, and processed and analyzed on the same day.

### 2.5. Biomarker Assessment

Serum peroxide concentrations were analyzed primarily as a continuous variable. For the exploratory group comparison, a data-derived cutoff of 2.70 nmol H_2_O_2_ equivalents/mL was used. This cutoff was used solely for descriptive exploratory purposes and was not intended as a clinically validated threshold.

Serum peroxide concentrations were measured using the ferrous oxidation–xylenol orange (FOX, PeroxiDetect™ Kit, Sigma-Aldrich, St. Louis, MO, USA) assay, which provides an aggregate measure of serum hydroperoxide burden through the oxidation of ferrous (Fe^2+^) to ferric (Fe^3+^) ions under acidic conditions, as previously described [19,20,21].

Ferric ions subsequently form a colored complex with xylenol orange, and absorbance was measured spectrophotometrically at 560 nm. Samples and assay reactions were protected from direct light during processing and analysis. The assay was performed at room temperature.

The assay does not distinguish individual peroxide species or their molecular sources, including lipid- and protein-derived hydroperoxides. Accordingly, serum peroxide values in the present study should be interpreted as an aggregate index of circulating hydroperoxide burden rather than as a measurement of a specific oxidative species or molecular source. Compared with markers of lipid peroxidation such as malondialdehyde or F2-isoprostanes, the FOX assay provides a broader but less specific estimate of circulating oxidative burden. Hydrogen peroxide standards were used for calibration, and results were expressed as nmol H_2_O_2_ equivalents/mL. Samples were analyzed immediately after serum separation, without freezing or storage. No predefined storage interval was used before peroxide measurement because the assay was initiated immediately after serum separation. Each sample was analyzed in duplicate, and the mean value was used for statistical analyses. Study-specific analytical performance characteristics, including the analytical range and intra-assay and inter-assay coefficients of variation, were not independently determined for the present study.

Baseline serum galectin-3 concentrations were measured at an external clinical laboratory (Bioclinica SA, Timișoara, Romania) using the Human Galectin-3 Quantikine ELISA kit (R&D Systems, Minneapolis, MN, USA; catalog no. DGAL30) on a DSX 4 analyzer. Results were reported in ng/mL. Manufacturer-reported intra-assay and inter-assay coefficients of variation for serum/plasma samples were 3.5–3.8% and 5.8–6.3%, respectively.

Baseline NT-proBNP concentrations were measured in serum in the hospital clinical laboratory using the VIDAS^®^ NT-proBNP2 (PBN2) assay (bioMérieux, Marcy-l’Étoile, France; Ref. 30458) on the VIDAS KUBE platform. The assay is an automated enzyme-linked fluorescent assay (ELFA) based on a sandwich immunoassay principle. Results were reported in pg/mL; the analytical measuring range was 15–25,000 pg/mL, with a limit of detection of 3.8 pg/mL and a limit of quantification of 4.9 pg/mL.

Baseline comorbidities, including obesity and chronic kidney disease, were recorded from the available medical documentation and were treated as documented clinical diagnoses rather than being derived from single baseline body mass index or eGFR measurements.

Routine laboratory variables used in the present analysis included serum uric acid, eGFR, absolute neutrophil count, and absolute lymphocyte count. Routine biochemical measurements, including serum creatinine and uric acid, were performed using a Dimension EXL 200 integrated chemistry system (Siemens Healthcare Diagnostics Inc., Newark, DE, USA). Complete blood counts, including absolute neutrophil and lymphocyte counts, were determined using a Sysmex XN-1000 hematology analyzer (Sysmex Corporation, Kobe, Japan). eGFR was calculated from serum creatinine using the 2021 CKD-EPI creatinine equation and expressed as mL/min/1.73 m^2^. The neutrophil-to-lymphocyte ratio (NLR) was calculated by dividing the absolute neutrophil count by the absolute lymphocyte count. Albuminuria was categorized according to the urinary albumin-to-creatinine ratio as A1 (<30 mg/g), A2 (30–300 mg/g), or A3 (>300 mg/g).

### 2.6. Echocardiographic Assessment

Transthoracic echocardiographic examinations were performed using a Vivid S5 ultrasound system (GE Healthcare, Horten, Norway). Left ventricular ejection fraction was calculated using the biplane Simpson method. Left ventricular end-diastolic volume was indexed to body surface area and reported as the left ventricular end-diastolic volume index (LVEDVi). Stroke volume was calculated as the difference between left ventricular end-diastolic volume and left ventricular end-systolic volume and was indexed to body surface area to derive the stroke volume index (SVI).

Left ventricular diastolic function was assessed using conventional transmitral Doppler and tissue Doppler parameters. The E/e′ ratio was calculated by dividing peak early transmitral flow velocity (E) by the mean of the septal and lateral early diastolic mitral annular velocities (e′).

Left ventricular global longitudinal strain (GLS) was assessed using two-dimensional speckle-tracking echocardiography from the apical four-chamber, two-chamber, and three-chamber views using EchoPAC software, version 203 (GE HealthCare, Horten, Norway). Automated endocardial tracking was visually inspected in each apical view, and contours were manually adjusted when tracking was considered inadequate. GLS was expressed as a percentage.

All transthoracic echocardiographic examinations and subsequent offline analyses were performed by the same investigator, who was blinded to serum peroxide, galectin-3, and NT-proBNP concentrations at the time of image analysis. No formal intra-observer or inter-observer reproducibility analysis was performed for LVEDVi or GLS.

### 2.7. Study Outcomes

The primary analysis evaluated the association between serum peroxide burden and NT-proBNP concentrations at baseline. Serum peroxide burden was analyzed as a continuous variable, and the primary association was assessed using Spearman rank correlation. Supporting regression analyses evaluated the relationship between serum peroxide burden and natural-log-transformed NT-proBNP before and after adjustment for age, sex, left ventricular ejection fraction, and estimated glomerular filtration rate.

Secondary correlation analyses evaluated the associations of galectin-3 with NT-proBNP, left ventricular end-diastolic volume index, and serum uric acid, as well as the associations of serum peroxide burden with galectin-3, absolute neutrophil count, and neutrophil-to-lymphocyte ratio.

Exploratory correlation analyses evaluated the associations of serum peroxide burden with global longitudinal strain and E/e′. Additional exploratory analyses included a joint biomarker model in which serum peroxide burden and galectin-3 were entered simultaneously as predictors of natural-log-transformed NT-proBNP. Biomarker concentrations were also compared across heart failure phenotypes. An additional exploratory comparison according to serum peroxide strata evaluated NT-proBNP concentrations in participants with serum peroxide levels ≤ 2.70 versus >2.70 nmol H_2_O_2_ equivalents/mL; this cutoff was data-derived and was not intended as a clinically validated threshold. Sensitivity analyses included partial rank correlations adjusted for eGFR for the associations of galectin-3 with NT-proBNP, LVEDVi, and serum uric acid; the galectin-3–NT-proBNP association was additionally evaluated after separate adjustment for atrial fibrillation and body mass index. An additional sensitivity analysis evaluated the exploratory joint biomarker model after inclusion of eGFR as a covariate.

### 2.8. Statistical Analysis

All statistical analyses were performed using MedCalc Statistical Software version 23.5.9 (MedCalc Software Ltd., Ostend, Belgium). Only baseline observations were included in the present analysis.

Distributional characteristics were evaluated using the Shapiro–Wilk test together with visual inspection of the data. Continuous variables are presented as mean ± standard deviation when approximately normally distributed and as median [interquartile range] otherwise. Categorical variables are presented as counts and percentages.

The primary association between serum peroxide burden and NT-proBNP was evaluated using Spearman rank correlation. Because NT-proBNP showed a right-skewed distribution, it was natural-log transformed for linear regression analyses. The unadjusted model included serum peroxide burden as the sole predictor. The first adjusted model additionally included age, sex, and left ventricular ejection fraction. An extended model further included estimated glomerular filtration rate. Regression coefficients obtained on the ln(NT-proBNP) scale were exponentiated when interpreted as percentage differences in NT-proBNP.

For multiplicity control, the six secondary and two exploratory Spearman correlations constituting the complete set of non-primary biomarker correlation analyses were treated as a single eight-test family. The primary peroxide–NT-proBNP association was prespecified and analyzed separately. The false discovery rate was controlled using the Benjamini–Hochberg procedure, and both nominal *p* values and adjusted q values are reported.

In the exploratory joint biomarker model, natural-log-transformed NT-proBNP was regressed simultaneously on serum peroxide burden and galectin-3. Model assumptions were assessed by examining residual distribution and residual-versus-fitted plots. Multicollinearity was evaluated using variance inflation factors. To evaluate the incremental explanatory contribution of the two biomarkers on an identical complete-case sample, two additional univariable linear regression models were fitted among the 29 participants with complete serum peroxide, galectin-3, and NT-proBNP data: one with serum peroxide burden alone and one with galectin-3 alone. These models were compared with the joint two-biomarker model using R^2^, adjusted R^2^, and nested partial F tests. These comparisons were considered exploratory. To assess potential renal confounding of the joint biomarker findings, an additional sensitivity model regressed ln(NT-proBNP) simultaneously on serum peroxide burden, galectin-3, and eGFR using complete cases for all four variables.

Sensitivity analyses were performed using partial correlations of ranked variables. The associations of galectin-3 with NT-proBNP, LVEDVi, and serum uric acid were evaluated after adjustment for ranked eGFR. The galectin-3–NT-proBNP association was additionally evaluated after separate adjustment for ranked atrial fibrillation status and ranked body mass index.

Serum peroxide, galectin-3, and NT-proBNP concentrations were compared across HFrEF, HFmrEF, and HFpEF using the Kruskal–Wallis test. Dunn’s pairwise procedure was performed only when the overall Kruskal–Wallis test was statistically significant.

NT-proBNP concentrations were compared between participants with serum peroxide levels ≤ 2.70 and >2.70 nmol H_2_O_2_ equivalents/mL using the Mann–Whitney U test. The Hodges–Lehmann location shift and its 95% confidence interval were also estimated.

Analyses were based on available complete cases for the variables included in each test. Missing values were not imputed. Extreme observations were retained unless a documented data-entry error was identified. All tests were two-sided, and a *p* value < 0.05 was considered statistically significant. Effect estimates are reported with 95% confidence intervals. Exploratory group comparisons and the sensitivity analyses were not included in the eight-test Benjamini–Hochberg family.

## 3. Results

### 3.1. Study Population

A total of 57 patients with heart failure were included in the baseline analysis. The median age was 71 [65–77] years, and 36 participants (63.2%) were men. Heart failure phenotype was available in 55 patients: 20 (36.4%) had HFrEF, 13 (23.6%) had HFmrEF, and 22 (40.0%) had HFpEF. Median NT-proBNP concentration was 1092 [802.5–2505.5] pg/mL. Serum peroxide measurements were available in 45 patients and galectin-3 measurements in 34 patients; analysis-specific sample sizes are reported for each analysis (Table 1). A flow diagram of participant inclusion and biomarker data availability is provided in Figure S3.

Among the 12 participants without serum peroxide measurements, the peroxide assay could not be performed on the day of sampling. Galectin-3 measurements were unavailable in 23 participants because galectin-3 testing was only available during part of the recruitment period. NT-proBNP measurements were unavailable in two participants because the test was not performed by the hospital laboratory.

In a comparison of participants included in the complete-case joint biomarker analysis (*n* = 29) with those with incomplete biomarker data (*n* = 28), age, sex, body mass index, NT-proBNP, and eGFR did not differ significantly between groups. However, complete-case participants had a higher mean LVEF (47.6 ± 10.4% vs. 41.5 ± 11.6%; *p* = 0.0458) and a lower prevalence of atrial fibrillation (31.0% vs. 60.7%; Fisher’s exact *p* = 0.0343). Detailed comparisons are provided in Table S1.

At baseline, most patients were receiving beta-blockers (87.7%) and statins (89.5%); 47.4% were treated with an ACE inhibitor, 19.3% with an angiotensin receptor blocker, 7.0% with an angiotensin receptor–neprilysin inhibitor, 50.9% with a mineralocorticoid receptor antagonist, and 66.7% with furosemide.

### 3.2. Primary Association Between Serum Peroxide Burden and NT-proBNP

Serum peroxide burden was positively correlated with NT-proBNP concentrations (Spearman’s ρ = 0.387, 95% CI 0.102–0.613; *p* = 0.0095; *n* = 44; Figure 1).

In an unadjusted linear regression model, serum peroxide burden was positively associated with ln(NT-proBNP) (B = 0.1844, 95% CI 0.0092–0.3596; *p* = 0.0396).

After adjustment for age, sex, and left ventricular ejection fraction, the association remained statistically significant (B = 0.1756, 95% CI 0.0067–0.3446; *p* = 0.0420; *n* = 43). This coefficient corresponds to an estimated 19.2% higher NT-proBNP concentration for each 1 nmol H_2_O_2_ equivalents/mL increase in serum peroxide burden.

In the extended model additionally adjusted for eGFR, the magnitude and direction of the association remained similar, although the 95% confidence interval included zero and the association did not reach statistical significance (B = 0.1646, 95% CI −0.0019 to 0.3310; *p* = 0.0525; *n* = 39). The extended model was statistically significant overall (model *p* = 0.0028) and had an adjusted R^2^ of 0.3198 (Table 2).

B represents the unstandardized regression coefficient for a 1 nmol H_2_O_2_ equivalents/mL increase in serum peroxide burden; the Spearman row reports ρ. For regression models, the reported *p* value refers to the serum peroxide coefficient. The age/sex/LVEF-adjusted model was statistically significant overall (F [4,38] = 3.188; *p* = 0.0236), as was the extended model additionally adjusted for eGFR (F [5,33] = 4.573; *p* = 0.0028). CI, confidence interval; eGFR, estimated glomerular filtration rate; LVEF, left ventricular ejection fraction; NT-proBNP, N-terminal pro-B-type natriuretic peptide.

### 3.3. Secondary Biomarker Associations

Galectin-3 was positively correlated with NT-proBNP (ρ = 0.476, 95% CI 0.159–0.704; *p* = 0.0051; qBH = 0.0320; *n* = 33), LVEDVi (ρ = 0.424, 95% CI 0.095–0.670; *p* = 0.0138; qBH = 0.0368; *n* = 33), and serum uric acid (ρ = 0.475, 95% CI 0.138–0.713; *p* = 0.0080; qBH = 0.0320; *n* = 30; Figure 2).

All three associations remained statistically significant after Benjamini–Hochberg false-discovery-rate correction. In sensitivity analyses, the associations of galectin-3 with NT-proBNP (partial rank r = 0.404; *p* = 0.0269; *n* = 31) and LVEDVi (partial rank r = 0.474; *p* = 0.0082; *n* = 31) remained statistically significant after adjustment for eGFR. The galectin-3–uric acid association also remained statistically significant after eGFR adjustment (partial rank r = 0.437; *p* = 0.0177; *n* = 30). Given the limited complete-case sample, this sensitivity analysis should be interpreted cautiously. The galectin-3–NT-proBNP association additionally remained statistically significant after separate adjustment for atrial fibrillation (partial rank r = 0.543; *p* = 0.0013; *n* = 33) and body mass index (partial rank r = 0.499; *p* = 0.0036; *n* = 33).

Serum peroxide burden was not significantly correlated with galectin-3 (ρ = 0.192; *p* = 0.3082), global longitudinal strain (ρ = 0.174; *p* = 0.2840), or E/e′ (ρ = −0.066; *p* = 0.6712).

Positive associations were observed between serum peroxide burden and both absolute neutrophil count (ρ = 0.293; *p* = 0.0601) and the neutrophil-to-lymphocyte ratio (ρ = 0.293; *p* = 0.0596); however, neither reached conventional statistical significance, and both remained nonsignificant after false-discovery-rate correction (qBH = 0.0962 for both; Table 3).

The FDR family comprised six secondary and two exploratory correlations (eight tests). The primary peroxide–NT-proBNP association was analyzed separately and was not included in this correction. CI, confidence interval; E/e′, ratio of early transmitral flow velocity to early diastolic mitral annular velocity; FDR, false discovery rate; GLS, global longitudinal strain; LVEDVi, left ventricular end-diastolic volume index; NLR, neutrophil-to-lymphocyte ratio; NT-proBNP, N-terminal pro-B-type natriuretic peptide; qBH, Benjamini–Hochberg-adjusted *p* value.

### 3.4. Joint Serum Peroxide and Galectin-3 Model

Among the 29 patients with complete serum peroxide, galectin-3, and NT-proBNP data, two univariable models and a joint two-biomarker model were evaluated using the identical complete-case sample.

In the peroxide-only model, serum peroxide burden was positively associated with ln(NT-proBNP) (B = 0.2506, 95% CI 0.0333–0.4680; *p* = 0.0254), with R^2^ = 0.1718 and adjusted R^2^ = 0.1411. In the galectin-3-only model, galectin-3 was positively associated with ln(NT-proBNP) (B = 0.1802, 95% CI 0.0893–0.2710; *p* = 0.0004), with R^2^ = 0.3802 and adjusted R^2^ = 0.3572.

When both biomarkers were entered simultaneously, serum peroxide burden (B = 0.1850, 95% CI 0.0041–0.3659; *p* = 0.0454) and galectin-3 (B = 0.1627, 95% CI 0.0753–0.2502; *p* = 0.0007) both remained significantly associated with ln(NT-proBNP). The joint model was statistically significant overall (F [2,26] = 11.5374; *p* = 0.0003), with R^2^ = 0.4702 and adjusted R^2^ = 0.4294. Addition of galectin-3 to the peroxide-only model increased R^2^ by 0.2984 (partial F = 14.6452; *p* = 0.0007), whereas addition of serum peroxide burden to the galectin-3-only model increased R^2^ by 0.0900 (partial F = 4.4184; *p* = 0.0454). Variance inflation factors were 1.039 for both predictors, indicating minimal collinearity. Given the limited complete-case sample (*n* = 29), these model comparisons were considered exploratory and the estimated R^2^ changes and partial F-test results should be interpreted cautiously because of limited model stability.

In an additional sensitivity model including eGFR (*n* = 27), serum peroxide burden (B = 0.2282, 95% CI 0.0462–0.4102; *p* = 0.0162) and galectin-3 (B = 0.1569, 95% CI 0.0617–0.2521; *p* = 0.0024) both remained significantly associated with ln(NT-proBNP). eGFR was not statistically significant in this model (B = −0.0159, 95% CI −0.0333 to 0.0016; *p* = 0.0725). The model had R^2^ = 0.5644 and adjusted R^2^ = 0.5076. Given the small complete-case sample, this analysis should be considered exploratory.

### 3.5. Biomarkers Across Heart Failure Phenotypes

Serum peroxide concentrations did not differ significantly among patients with HFrEF, HFmrEF, and HFpEF (Kruskal–Wallis H = 2.635; *p* = 0.2678). Galectin-3 concentrations also did not differ across heart failure phenotypes (H = 0.269; *p* = 0.8744).

In contrast, NT-proBNP concentrations differed significantly across the three phenotypes (H = 7.225; *p* = 0.0270; Figure S2). Dunn’s post hoc analysis identified significantly higher NT-proBNP concentrations in patients with HFrEF than in those with HFmrEF, whereas comparisons involving HFpEF were not statistically significant. Given the small phenotype-specific sample sizes, the absence of statistically significant differences in serum peroxide or galectin-3 concentrations should not be interpreted as evidence of equivalence across heart failure phenotypes.

### 3.6. Exploratory Analysis According to Serum Peroxide Strata

In the exploratory analysis according to serum peroxide strata, patients with serum peroxide concentrations > 2.70 nmol H_2_O_2_ equivalents/mL had significantly higher NT-proBNP concentrations than those with concentrations ≤ 2.70 nmol H_2_O_2_ equivalents/mL (2237.5 [982.5–3668.5] versus 874 [472.5–1339.5] pg/mL; Mann–Whitney U = 138; exact *p* = 0.0157; Figure S1). This cutoff was used solely for descriptive exploratory purposes and should not be interpreted as a clinically validated threshold.

The Hodges–Lehmann estimated location shift for the higher-peroxide group relative to the lower-peroxide group was 872 pg/mL (95% CI 129–2083 pg/mL).

The main exploratory analyses are summarized in Table 4.

Same-sample biomarker models were fitted in the identical 29 complete cases. In the joint model, addition of galectin-3 to the peroxide-only model increased R^2^ by 0.2984 (partial F = 14.6452; *p* = 0.0007), whereas addition of serum peroxide burden to the galectin-3-only model increased R^2^ by 0.0900 (partial F = 4.4184; *p* = 0.0454). Variance inflation factors were 1.039 for both predictors. Exploratory group comparisons were not included in the eight-test Benjamini–Hochberg family. B, unstandardized regression coefficient; CI, confidence interval; eGFR, estimated glomerular filtration rate; HFmrEF, heart failure with mildly reduced ejection fraction; HFpEF, heart failure with preserved ejection fraction; HFrEF, heart failure with reduced ejection fraction; NT-proBNP, N-terminal pro-B-type natriuretic peptide.

## 4. Discussion

### 4.1. Principal Findings

The principal finding of this baseline analysis was that higher serum peroxide burden was associated with higher NT-proBNP concentrations in patients with chronic heart failure. This relationship remained statistically significant after adjustment for age, sex, and left ventricular ejection fraction. After additional adjustment for eGFR, the magnitude and direction of the association remained similar, although the 95% confidence interval included zero and statistical significance was not reached.

Galectin-3 showed a distinct pattern of associations, correlating positively with NT-proBNP, LVEDVi, and serum uric acid. These relationships remained significant after false-discovery-rate correction. Furthermore, both serum peroxide burden and galectin-3 retained statistically significant associations with ln-transformed NT-proBNP when entered simultaneously into the same exploratory model, with minimal collinearity. However, serum peroxide burden was not significantly correlated with galectin-3.

Taken together, these findings indicate different observed cross-sectional association patterns for serum peroxide burden and galectin-3 within this cohort. The absence of a statistically significant correlation between the two biomarkers, together with their concurrent associations with NT-proBNP in the exploratory joint model, should not be interpreted as evidence of biological independence or of separate mechanistic pathways. Given the cross-sectional design and limited sample size, these findings are hypothesis-generating and do not establish temporal, causal, or mechanistic relationships.

### 4.2. Serum Peroxide Burden and NT-proBNP

The observed association between serum peroxide burden and NT-proBNP was consistent with a cross-sectional relationship between higher circulating oxidative burden and greater heart-failure-related hemodynamic stress in this cohort. Previous human studies have reported higher circulating oxidative-stress markers in chronic heart failure than in control populations [22,23,24]. In one small human study, plasma total peroxide concentrations and several leukocyte markers of oxidative-nitrative stress were elevated in patients with chronic heart failure, while pro-BNP concentrations correlated positively with plasma peroxide levels, the oxidative stress index, and leukocyte poly(ADP-ribose) polymerase (PARP) activation [12]. More recent data from a large heart failure cohort further support this relationship, as greater systemic oxidative stress, reflected by lower circulating free thiol concentrations, was associated with higher NT-proBNP levels and greater heart failure severity [25]. The present findings are directionally consistent with these larger data, although direct comparison should be cautious because the oxidative-stress marker, study population, and analytical approach differed, and our sample was substantially smaller.

In the present cohort, the association between serum peroxide burden and NT-proBNP remained statistically significant after adjustment for age, sex, and LVEF. The corresponding regression coefficient indicated an estimated 19.2% higher NT-proBNP concentration for each 1 nmol H_2_O_2_ equivalents/mL higher serum peroxide concentration. This estimate represents a cross-sectional statistical association and should not be interpreted as a causal effect or as a clinically validated change, because no established clinically meaningful threshold for serum peroxide concentration is available in this setting. After additional adjustment for eGFR, the regression coefficient remained positive and of similar magnitude, but the confidence interval included zero and the association no longer reached conventional statistical significance. Therefore, the present data do not establish that the peroxide–NT-proBNP association is independent of renal function; the attenuation may reflect renal confounding, reduced statistical precision in the smaller complete-case sample, or both.

The exploratory stratified comparison was directionally consistent with the continuous analysis: patients with serum peroxide concentrations above 2.70 nmol H_2_O_2_ equivalents/mL had substantially higher NT-proBNP concentrations. The continuous analysis remains preferable, as dichotomization necessarily reduces information and statistical power.

Serum peroxide burden should not be interpreted as a direct measurement of myocardial reactive oxygen species. The FOX assay estimates circulating peroxide burden through the oxidation of ferrous to ferric ions, followed by detection with xylenol orange, with results expressed as nmol H_2_O_2_ equivalents/mL [19,20,21]. Therefore, it provides an integrated systemic redox signal that may be influenced by several organs, metabolic pathways, medications, inflammatory activity, and antioxidant capacity.

### 4.3. Galectin-3, NT-proBNP, and Left Ventricular Structural Phenotype

Galectin-3 was positively associated with both NT-proBNP and LVEDVi. Its association with NT-proBNP also suggests a cross-sectional relationship with greater hemodynamic burden, although the present data do not establish that the two biomarkers represent biologically distinct pathways. These cross-sectional associations are compatible with the proposed involvement of galectin-3 in myocardial fibrosis, extracellular matrix turnover, and remodeling-related processes, but they do not demonstrate ongoing ventricular remodeling.

Previous clinical data support an association between circulating galectin-3 and ventricular remodeling. In a cohort of 240 patients with severe chronic heart failure, baseline galectin-3 was associated with changes in left ventricular end-diastolic volume over three months and with long-term mortality. Patients whose LVEDV decreased had lower baseline galectin-3 concentrations than those with stable or increasing LVEDV [26]. More recent data have similarly demonstrated positive associations between circulating galectin-3 and LVEDV and LVESV in patients with HFrEF [27].

Other chronic heart failure studies have also reported associations between circulating galectin-3 and indices of left ventricular structure and function, including LV end-diastolic dimensions and volumes and LVEF, as well as functional capacity and adverse clinical outcomes [27,28,29]. Accordingly, the galectin-3 associations observed in our smaller exploratory cohort are broadly consistent with findings from larger chronic heart failure studies, but the present study should be considered supportive rather than definitive evidence. Serial galectin-3 measurements may provide incremental prognostic information beyond a single baseline measurement [30,31], although this cannot yet be assessed in the present baseline analysis.

The observed correlation with LVEDVi is therefore biologically plausible, although circulating galectin-3 should not be regarded as a direct measure of myocardial fibrosis. Rather, circulating galectin-3 may reflect fibro-inflammatory processes associated with adverse cardiac remodeling [7]. Its circulating concentration is influenced by inflammation, age, comorbidities, and renal handling, which limits its cardiac specificity [31,32,33]. In sensitivity analyses, the associations of galectin-3 with NT-proBNP and LVEDVi remained statistically significant after adjustment for eGFR, suggesting that these relationships were not entirely explained by renal function in this cohort. However, residual renal and other clinical confounding cannot be excluded. The galectin-3–NT-proBNP association also remained statistically significant after separate adjustment for atrial fibrillation and body mass index, indicating that neither factor alone accounted for the observed association, although residual confounding remains possible.

### 4.4. Galectin-3, Uric Acid, and the Cardiorenal Axis

The association between galectin-3 and uric acid places our findings within the broader cardiorenal context of heart failure, where renal dysfunction, oxidative stress, inflammation, and fibrosis frequently coexist [34,35].

This relationship remained statistically significant after rank-based adjustment for eGFR, suggesting that it was not entirely attributable to differences in estimated glomerular filtration. However, residual renal and metabolic confounding cannot be excluded.

Previous studies in chronic heart failure have demonstrated associations of circulating galectin-3 with uric acid, cystatin C, eGFR, and markers of oxidative stress [31,36,37]. In addition, impaired renal handling may contribute to higher circulating galectin-3 concentrations in heart failure, particularly in patients with renal dysfunction [32,33,38].

Accordingly, the observed galectin-3–uric acid association may reflect a shared cardiorenal, metabolic, and fibro-inflammatory milieu rather than a direct biological relationship between the two biomarkers [36,39,40]. Although the association remained statistically significant after adjustment for eGFR, this sensitivity analysis included only 30 participants and residual renal and metabolic confounding remains possible. Serum uric acid concentrations in heart failure may additionally be influenced by congestion, renal handling, diuretic therapy, diet, and xanthine oxidase activity [41]. Medication-related confounding was not specifically accounted for in this analysis and should therefore be considered when interpreting this finding.

### 4.5. Concurrent Associations of Serum Peroxides and Galectin-3

Serum peroxide burden and galectin-3 were not significantly correlated with each other, while both were associated with ln(NT-proBNP) when included simultaneously in the exploratory joint model. Variance inflation factors were close to 1, indicating minimal statistical collinearity between the two predictors. However, the absence of a significant correlation between the biomarkers does not establish biological independence or demonstrate that they represent distinct mechanistic pathways. The observed pattern is compatible with the possibility that serum peroxide burden and galectin-3 are associated with different aspects of the heart-failure phenotype, but this interpretation remains exploratory. This pattern is compatible with different cross-sectional association profiles rather than demonstrating biological complementarity. Multimarker studies have previously shown that biomarkers representing different pathophysiological domains may provide non-redundant information in heart failure [42,43]. Serum peroxide concentrations provide an index of circulating oxidative burden, whereas galectin-3 has been associated with macrophage activation, fibro-inflammatory processes, extracellular-matrix remodeling, and cardiorenal interactions [15,33,44]. In the 29 complete cases, serum peroxides alone explained 17.2% of the variance in ln(NT-proBNP), galectin-3 38.0%, and the joint model 47.0%.

Adding galectin-3 to the peroxide-only model increased R^2^ by 0.2984 (*p* = 0.0007), whereas adding serum peroxide burden to the galectin-3-only model increased R^2^ by 0.0900 (*p* = 0.0454).

On the identical complete-case sample, each biomarker added explanatory information to a model containing the other, although the contribution of galectin-3 was larger. Because these comparisons were exploratory and based on only 29 participants, the R^2^ changes and partial F-test estimates may be unstable and should not be interpreted as evidence of incremental clinical or prognostic performance.

In a sensitivity analysis additionally including eGFR, both serum peroxide burden and galectin-3 remained significantly associated with ln(NT-proBNP). However, this model included only 27 participants and should therefore be interpreted cautiously as an exploratory assessment of renal confounding rather than evidence of independent clinical value.

A graphical summary of the main observed associations is provided in Figure 3.

### 4.6. Associations with Inflammatory and Echocardiographic Parameters

Serum peroxide burden was not significantly associated with either absolute neutrophil count or NLR, and neither correlation was statistically significant after false-discovery-rate correction. Accordingly, the present data do not provide sufficient evidence for an association between circulating peroxide burden and these inflammatory indices.

No significant relationships were identified between serum peroxide burden and either GLS or E/e′. Circulating peroxide burden may not closely reflect myocardial oxidative stress, and both GLS and E/e′ are influenced by loading conditions, technical factors, and the underlying cardiac phenotype [45,46,47]. E/e′ may additionally vary with hemodynamic conditions such as heart rate, blood pressure, and volume status, which were not specifically incorporated into the present association analysis. In addition, the available sample sizes may have been insufficient to detect modest associations.

These negative findings do not exclude weaker relationships with myocardial mechanics or diastolic function that this sample may have been underpowered to detect.

### 4.7. Heart Failure Phenotypes

Serum peroxide and galectin-3 concentrations did not differ significantly across HFrEF, HFmrEF, and HFpEF in the present cohort. However, the phenotype-specific subgroups were small, and the absence of statistically significant between-group differences should not be interpreted as evidence of biomarker equivalence across the LVEF spectrum. These findings therefore do not exclude biological differences in oxidative or fibro-inflammatory pathways among heart failure phenotypes [48,49,50].

The phenotype subgroups were small, and the present analysis was not powered to establish biomarker equivalence. In contrast, NT-proBNP concentrations differed significantly across phenotypes, with higher values in HFrEF than in HFmrEF. Because comparisons involving HFpEF were not significant, the NT-proBNP pattern should not be interpreted as a simple gradient across LVEF categories.

Factors such as atrial fibrillation, age, renal function, obesity, congestion, and treatment may influence NT-proBNP independently of LVEF and may have contributed to the overlap observed among the three phenotypes [51,52].

### 4.8. Clinical and Research Implications

The different association patterns observed for serum peroxides, galectin-3, and NT-proBNP are consistent with a multidimensional biomarker framework in heart failure [6,53], but the present data do not establish that these markers represent biologically distinct domains.

The present data, however, are insufficient to determine whether measuring serum peroxides or galectin-3 improves diagnosis, prognostic discrimination, or therapeutic decision-making beyond established clinical variables and natriuretic peptides. No clinical outcome analysis, discrimination analysis, reclassification analysis, or externally validated cutoff was performed. Accordingly, the present findings do not support a clinical decision algorithm incorporating serum peroxide burden or galectin-3 at this stage. Such an approach would require validation in larger prospective cohorts with clinical outcomes and predefined biomarker thresholds.

Because the present analysis includes baseline data only, it cannot determine whether serum peroxide burden or galectin-3 changes with treatment or disease progression.

The prospective longitudinal component of the parent cohort provides an opportunity to test whether changes in serum peroxide burden and galectin-3 after SGLT2 inhibitor initiation are associated with longitudinal changes in NT-proBNP, ventricular structural and functional parameters, endothelial function, and exercise capacity [54,55]. Specific testable hypotheses include whether reductions in serum peroxide burden are associated with reductions in NT-proBNP and whether changes in galectin-3 are associated with changes in cardiac structural or cardiorenal parameters. Such analyses may help determine whether the baseline associations observed here persist longitudinally or change in parallel with treatment-related phenotypic changes.

### 4.9. Strengths and Limitations

Strengths of the present study include the evaluation of patients across the LVEF spectrum, integration of biochemical and echocardiographic variables, the clear distinction between the main peroxide–NT-proBNP analysis and the secondary and exploratory analyses, adjustment for clinically relevant covariates, control of the false discovery rate for the secondary correlation family, and sensitivity analysis of the galectin-3–uric acid association after adjustment for eGFR. The use of identical complete-case samples for direct comparison of the single- and two-biomarker models further strengthened the exploratory comparison of their concurrent associations with NT-proBNP.

Several limitations should be acknowledged.

First, the cross-sectional design precludes conclusions regarding temporality or causality and does not permit assessment of biomarker changes with treatment or disease progression. The number and characteristics of eligible patients who declined participation were not systematically recorded. Consequently, the participation rate could not be calculated, and selection bias related to voluntary participation cannot be excluded. Exclusion of patients with eGFR < 30 mL/min/1.73 m^2^ limits the generalizability of the findings to patients with more advanced renal dysfunction. Exclusion of patients with active systemic inflammatory disease, acute infection, or sepsis further limits generalizability to heart failure populations with these conditions, in whom inflammatory and oxidative biomarker profiles may differ. In addition, inclusion was restricted to patients considered suitable for SGLT2 inhibitor therapy, which may further limit generalizability to the broader chronic heart failure population.

Second, this was a single-center study with a relatively small cohort, and several analyses involved substantially smaller complete-case samples. In particular, the exploratory joint biomarker model included only 29 participants, limiting statistical precision and model stability and increasing the risk of overfitting.

Third, missing data were handled using complete-case analysis without imputation. Missing measurements resulted from assay unavailability on the day of sampling, limited availability of galectin-3 testing during the recruitment period, or absence of NT-proBNP determination in the hospital laboratory. Because biomarker and clinical-variable availability differed across participants, selection bias may have been introduced if missingness was not random. The complete-case sample had a higher mean LVEF and a lower prevalence of atrial fibrillation than participants with incomplete biomarker data, indicating that complete-case selection may have influenced the composition of the exploratory joint-model sample.

Fourth, serum peroxide burden reflects systemic oxidative status but cannot distinguish myocardial from extracardiac sources of oxidative stress [56,57,58].

Fifth, single baseline biomarker measurements may be affected by biological variability, pre-analytical and analytical factors, and concomitant treatment. The FOX assay provides an aggregate estimate of circulating hydroperoxide burden and does not distinguish individual peroxide species, lipid- versus protein-derived hydroperoxides, or myocardial from extracardiac sources. Immediate analysis without freezing minimized storage-related variability within the study, but this pre-analytical protocol may limit direct comparability with studies using stored or differently processed samples. Study-specific analytical performance characteristics of the FOX assay, including the analytical range and intra-assay and inter-assay precision, were not independently determined.

Sixth, all echocardiographic examinations and offline analyses were performed by a single investigator, and formal intra-observer and inter-observer reproducibility analyses were not performed. This may limit the reproducibility of measurements such as LVEDVi and GLS.

Seventh, residual confounding remains possible despite adjustment for age, sex, LVEF, and eGFR; other potentially relevant factors, including atrial fibrillation, body mass index, congestion, heart failure phenotype, and concomitant medication use, were not comprehensively incorporated into all models. Medication-related confounding, including potential effects of diuretic and urate-modifying therapies, could not be fully accounted for in the present analyses. In addition, obesity and chronic kidney disease were based on documented clinical diagnoses rather than independently adjudicated study definitions, and some degree of comorbidity misclassification therefore cannot be excluded. Given the small complete-case samples, simultaneous adjustment for all potentially relevant clinical and treatment-related covariates was not attempted because this would have resulted in an overparameterized and potentially unstable model.

Eighth, some analyses—including the joint biomarker model, phenotype comparisons, data-derived peroxide stratification, and partial rank correlation—were exploratory. The joint biomarker model, in particular, was based on only 29 complete cases, limiting statistical precision and model stability and increasing the risk of overfitting. No internal validation, bootstrap analysis, or external validation of the joint biomarker model was performed.

These findings require confirmation in larger independent cohorts. Finally, the present analysis did not evaluate hospitalization, mortality, or incremental prognostic performance.

## 5. Conclusions

In this cross-sectional baseline analysis of patients with chronic heart failure, higher serum peroxide burden was associated with higher NT-proBNP concentrations, although this association no longer reached conventional statistical significance after additional adjustment for eGFR. Galectin-3 was positively associated with NT-proBNP, LVEDVi, and serum uric acid.

Serum peroxide burden and galectin-3 were not significantly correlated with each other, yet both retained statistically significant associations with ln-transformed NT-proBNP in an exploratory joint model.

Together, these results indicate different cross-sectional association patterns for serum peroxide burden and galectin-3, without establishing biological independence, mechanistic distinction, or clinical complementarity.

Given the limited sample size and cross-sectional design, these findings should be considered hypothesis-generating and require confirmation in larger longitudinal cohorts. The present study does not establish prognostic value beyond NT-proBNP.

## Figures and Tables

**Figure 1:**
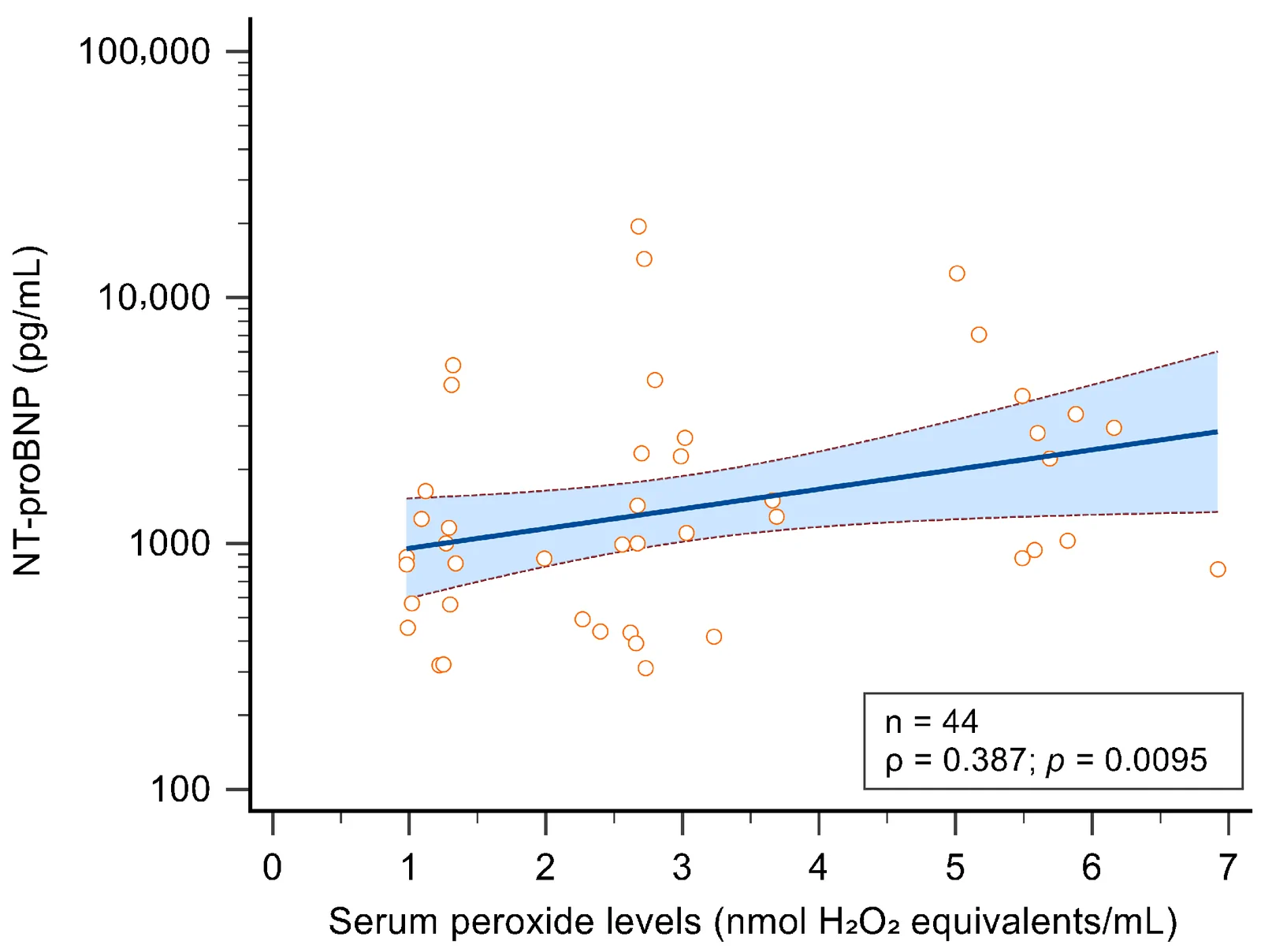
Association between serum peroxide levels and NT-proBNP concentrations in patients with heart failure. Orange open circles represent individual patients. The reported ρ and *p* value correspond to Spearman rank correlation. The solid blue line represents a fitted relationship displayed for visualization only and was not used for statistical inference. The light-blue shaded area, bounded by red dashed lines, represents the 95% confidence band. NT-proBNP is displayed on a logarithmic scale. NT-proBNP, N-terminal pro-B-type natriuretic peptide.

**Figure 2:**
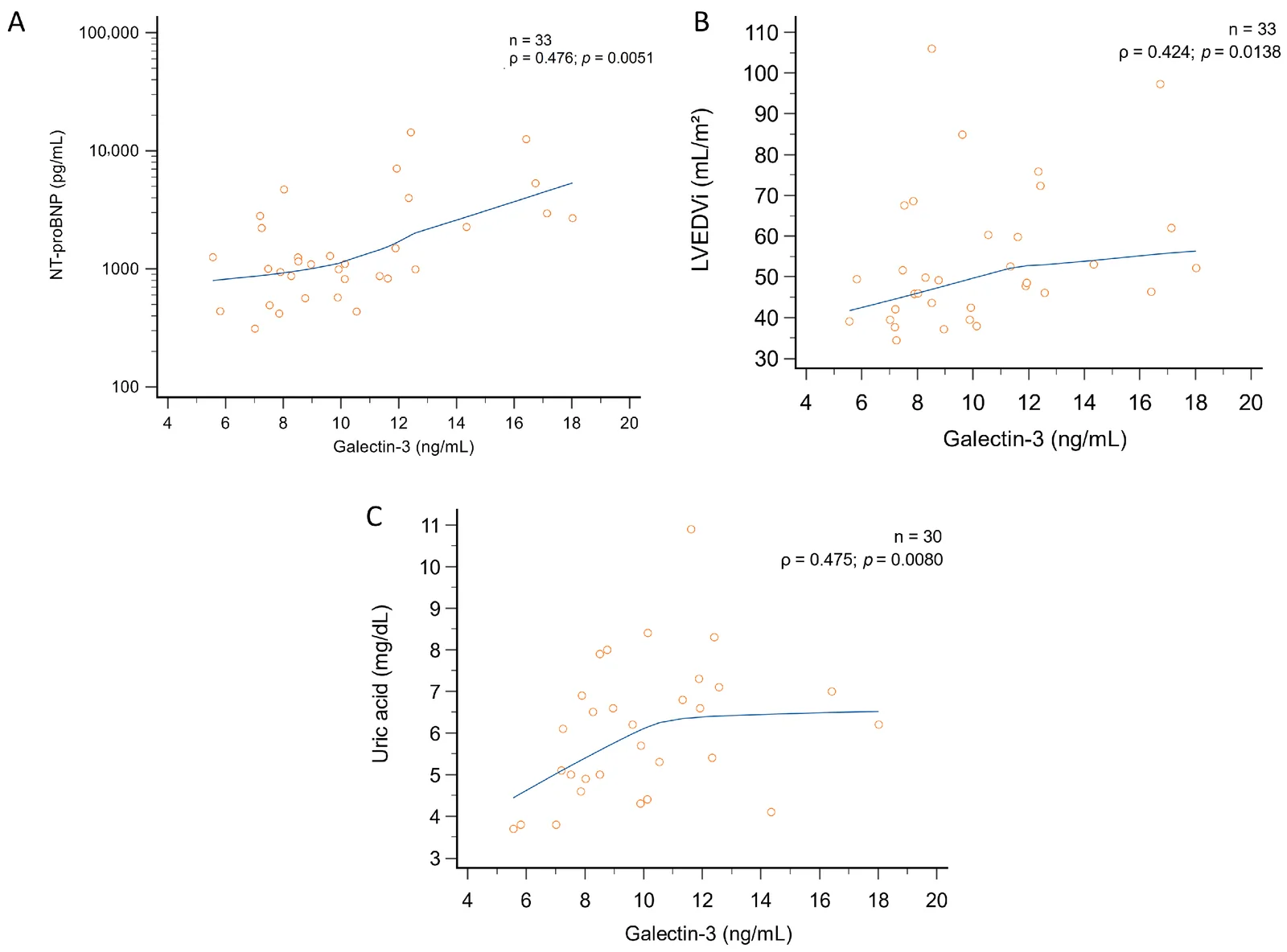
Associations of galectin-3 with markers of heart failure severity, left ventricular structure, and cardiorenal dysfunction. (**A**) Galectin-3 and NT-proBNP; (**B**) galectin-3 and left ventricular end-diastolic volume index (LVEDVi); (**C**) galectin-3 and serum uric acid. Reported ρ and *p* values correspond to Spearman rank correlations. Orange open circles represent individual patients, and light-blue lines represent LOWESS curves. LOWESS curves are displayed for descriptive visualization only and were not used for statistical inference. LOWESS, locally weighted scatterplot smoothing; NT-proBNP, N-terminal pro-B-type natriuretic peptide.

**Figure 3:**
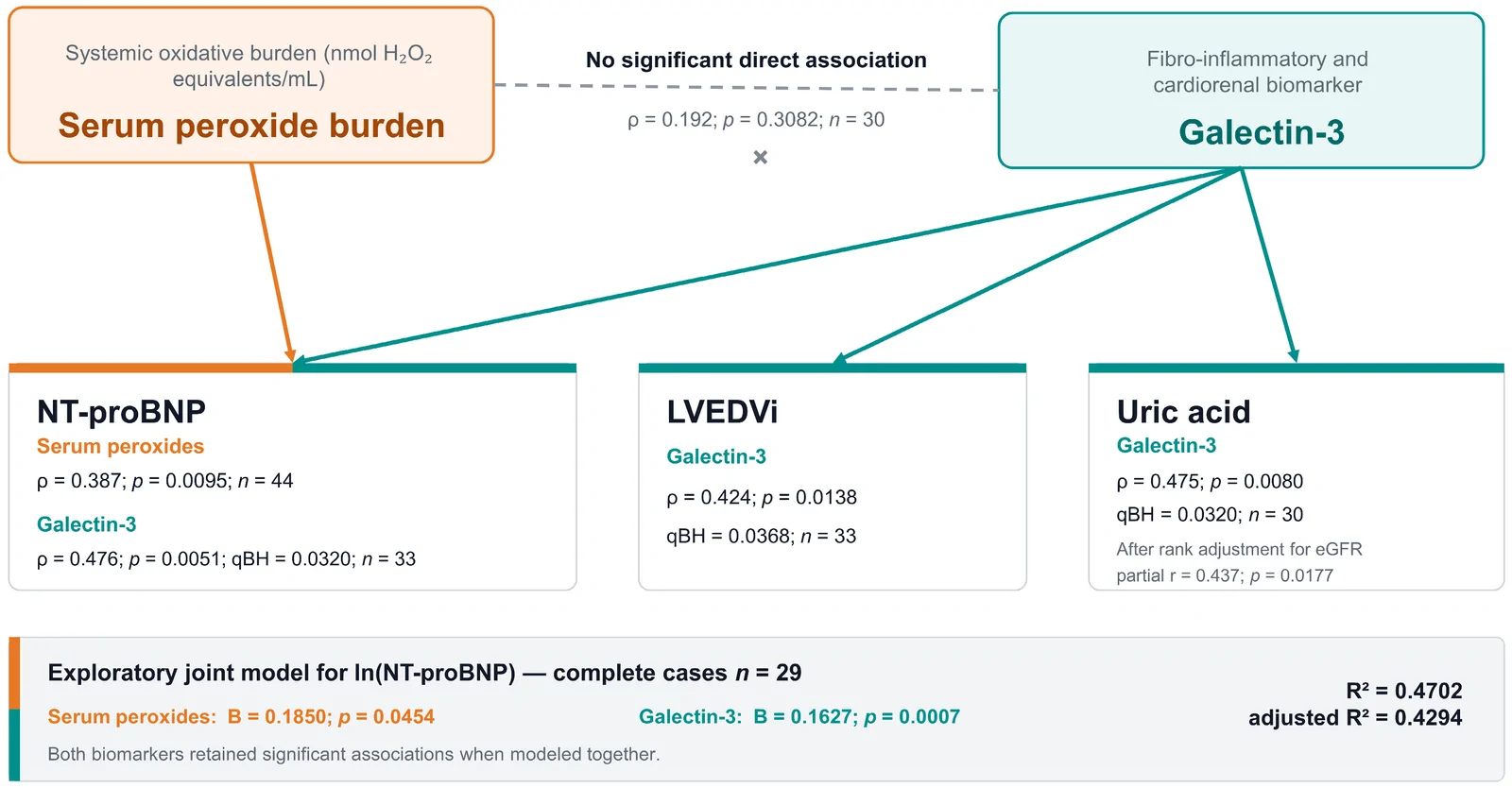
Graphical summary of the main observed baseline associations of serum peroxide burden and galectin-3 in patients with chronic heart failure. Solid arrows indicate statistically significant positive associations, whereas the dashed line indicates that no statistically significant direct association was observed between serum peroxide burden and galectin-3. In the exploratory complete-case joint model, both biomarkers retained statistically significant associations with ln(NT-proBNP). Secondary galectin-3 associations remained significant after Benjamini–Hochberg false-discovery-rate correction. The arrows summarize observed statistical associations and should not be interpreted as indicating causal relationships. All measurements were obtained at baseline, before SGLT2 inhibitor initiation. eGFR, estimated glomerular filtration rate; LVEDVi, left ventricular end-diastolic volume index; NT-proBNP, N-terminal pro-B-type natriuretic peptide; qBH, Benjamini–Hochberg-adjusted *p* value; SGLT2, sodium–glucose cotransporter-2.

**Table 1:** Baseline characteristics of the overall cohort.

Characteristic	Available *n*	Overall Cohort
**Demographics and hemodynamics**		
Age, years	57	71 [65–77]
Male sex	57	36 (63.2%)
Body mass index, kg/m^2^	57	28.5 [25.5–32.0]
Systolic blood pressure, mmHg	56	130 [110–145]
Diastolic blood pressure, mmHg	56	80 [70–80]
**Comorbidities**		
Diabetes mellitus	57	12 (21.1%)
Hypertension	57	43 (75.4%)
Hypercholesterolemia	57	40 (70.2%)
Current smoking	57	8 (14.0%)
Previous stroke/transient ischemic attack	57	11 (19.3%)
Atrial fibrillation	57	26 (45.6%)
Coronary artery disease	57	25 (43.9%)
Documented obesity	57	27 (47.4%)
Documented chronic kidney disease	52	24 (46.2%)
**Baseline medication**		
Beta-blocker	57	50 (87.7%)
Statin	57	51 (89.5%)
ACE inhibitor	57	27 (47.4%)
Angiotensin receptor blocker	57	11 (19.3%)
Angiotensin receptor–neprilysin inhibitor	57	4 (7.0%)
Mineralocorticoid receptor antagonist	57	29 (50.9%)
Furosemide	57	38 (66.7%)
Calcium-channel blocker	57	10 (17.5%)
Aspirin/dual antiplatelet therapy	57	24 (42.1%)
Oral anticoagulant (DOAC/VKA)	57	37 (64.9%)
Ivabradine	57	1 (1.8%)
**Functional status and heart failure phenotype**		
NYHA class II	57	42 (73.7%)
NYHA class III	57	14 (24.6%)
NYHA class IV	57	1 (1.8%)
HFrEF	55	20 (36.4%)
HFmrEF	55	13 (23.6%)
HFpEF	55	22 (40.0%)
**Biomarkers**		
NT-proBNP, pg/mL	55	1092 [802.5–2505.5]
Serum peroxide burden, nmol H_2_O_2_ equivalents/mL	45	2.67 [1.31–3.69]
Galectin-3, ng/mL	34	9.75 [7.87–11.92]
**Renal and inflammatory profile**		
eGFR, mL/min/1.73 m^2^	52	65.3 ± 17.7
Uric acid, mg/dL	52	6.1 ± 1.7
Albuminuria A1 (<30 mg/g)	51	19 (37.3%)
Albuminuria A2 (30–300 mg/g)	51	17 (33.3%)
Albuminuria A3 (>300 mg/g)	51	15 (29.4%)
Neutrophils, ×10^9^/L	54	4.93 [3.94–5.74]
Lymphocytes, ×10^9^/L	54	1.85 [1.38–2.36]
Neutrophil-to-lymphocyte ratio	54	2.71 [1.83–3.60]
**Echocardiographic profile**		
Left ventricular ejection fraction, %	55	44.6 ± 11.3
LV end-diastolic volume index, mL/m^2^	55	49.5 [42.3–60.4]
Stroke volume index, mL/m^2^	55	22.8 [17.8–27.5]
E/e′ ratio	55	9.8 [7.1–12.6]
Global longitudinal strain, %	46	−12.1 ± 4.1

Data are presented as mean ± standard deviation, median [interquartile range], or *n* (%), as appropriate. Percentages were calculated using the available denominator shown in the middle column. HF phenotypes were classified as HFrEF (LVEF ≤ 40%), HFmrEF (LVEF 41–49%), or HFpEF (LVEF ≥ 50%). eGFR, estimated glomerular filtration rate; LVEF, left ventricular ejection fraction; NT-proBNP, N-terminal pro-B-type natriuretic peptide; NYHA, New York Heart Association; E/e′, ratio of early transmitral flow velocity to early diastolic mitral annular velocity.

**Table 2:** Primary peroxide–NT-proBNP association and supporting regression models.

Analysis/Model	*n*	Effect Estimate (95% CI)	*p*	Adjusted R^2^
Primary Spearman correlation	44	ρ = 0.387 (0.102 to 0.613)	0.0095	-
Unadjusted ln(NT-proBNP) model	44	B = 0.1844 (0.0092 to 0.3596)	0.0396	0.0755
Adjusted for age, sex, and LVEF	43	B = 0.1756 (0.0067 to 0.3446)	0.0420	0.1724
Additionally adjusted for eGFR	39	B = 0.1646 (−0.0019 to 0.3310)	0.0525	0.3198

**Table 3:** Secondary and exploratory Spearman correlations with Benjamini–Hochberg correction.

Association	*n*	Spearman ρ (95% CI)	*p*	qBH	Significant After FDR Correction
Galectin-3—NT-proBNP	33	0.476 (0.159 to 0.704)	0.0051	0.0320	Yes
Peroxides—galectin-3	30	0.192 (−0.180 to 0.517)	0.3082	0.3522	No
Galectin-3—LVEDVi	33	0.424 (0.095 to 0.670)	0.0138	0.0368	Yes
Galectin-3—uric acid	30	0.475 (0.138 to 0.713)	0.0080	0.0320	Yes
Peroxides—neutrophils	42	0.293 (−0.013 to 0.548)	0.0601	0.0962	No
Peroxides—NLR	42	0.293 (−0.012 to 0.548)	0.0596	0.0962	No
Peroxides—GLS	40	0.174 (−0.146 to 0.460)	0.2840	0.3522	No
Peroxides—E/e′	44	−0.066 (−0.356 to 0.236)	0.6712	0.6712	No

**Table 4:** Exploratory analyses.

Analysis	*n*	Main Result	*p*
Peroxide-only model, complete-case sample	29	B = 0.2506 (95% CI 0.0333–0.4680); R^2^ = 0.1718; adjusted R^2^ = 0.1411	0.0254
Galectin-3-only model, complete-case sample	29	B = 0.1802 (95% CI 0.0893–0.2710); R^2^ = 0.3802; adjusted R^2^ = 0.3572	0.0004
Joint biomarker model, complete-case sample	29	serum peroxides: B = 0.1850 (95% CI 0.0041–0.3659); galectin-3: B = 0.1627 (95% CI 0.0753–0.2502); R^2^ = 0.4702; adjusted R^2^ = 0.4294; ΔR^2^ = +0.2984 vs. peroxides alone and +0.0900 vs. galectin-3 alone	0.0454 (peroxides); 0.0007 (galectin-3)
Galectin-3–uric acid partial rank correlation adjusted for eGFR	30	Partial rank r = 0.437	0.0177
Peroxides by HF phenotype	44	HFrEF 2.710 [1.310–5.170] (*n* = 18); HFmrEF 3.175 [2.400–5.690] (*n* = 10); HFpEF 2.415 [1.195–2.875] (*n* = 16); H = 2.635	0.2678
Galectin-3 by HF phenotype	33	HFrEF 9.93 [7.90–12.42] (*n* = 10); HFmrEF 9.62 [7.68–11.90] (*n* = 7); HFpEF 9.33 [7.51–11.76] (*n* = 16); H = 0.269	0.8744
NT-proBNP by HF phenotype	53	HFrEF 2321 [955.5–4677.3] (*n* = 19); HFmrEF 941 [423.5–1517.3] (*n* = 13); HFpEF 1001 [757.8–1440.8] (*n* = 21); H = 7.225; Dunn: HFrEF > HFmrEF only	0.0270
NT-proBNP: peroxide ≤2.7 vs. >2.7 nmol H_2_O_2_ equivalents/mL	44	874 [472.5–1339.5] vs. 2237.5 [982.5–3668.5] pg/mL; U = 138; Hodges-Lehmann difference = 872 (129 to 2083)	0.0157

## Data Availability

The data presented in this study are available from the corresponding author upon reasonable request and subject to applicable ethical and institutional requirements. The data are not publicly available due to privacy and ethical restrictions related to clinical participant data.

## Supplementary Materials

The following supporting information can be downloaded at: https://www.mdpi.com/article/10.3390/biomedicines14102166/s1, Figure S1: NT-proBNP concentrations by serum peroxide group; Figure S2: NT-proBNP concentrations across heart failure phenotypes; Figure S3: Flow diagram of participant inclusion and biomarker data availability for the main analyses. Table S1: Comparison of baseline characteristics between participants included in the complete-case joint biomarker analysis and those with incomplete biomarker data.

## Abbreviations

The following abbreviations are used in this manuscript:

APC	article processing charge
CI	confidence interval
CKD-EPI	Chronic Kidney Disease Epidemiology Collaboration
E/e′	ratio of early transmitral flow velocity to early diastolic mitral annular velocity
eGFR	estimated glomerular filtration rate
ESC	European Society of Cardiology
FDR	false discovery rate
FOX	ferrous oxidation–xylenol orange assay
GLS	global longitudinal strain
HF	heart failure
HFrEF	heart failure with reduced ejection fraction
HFmrEF	heart failure with mildly reduced ejection fraction
HFpEF	heart failure with preserved ejection fraction
LV	left ventricular
LVEDV	left ventricular end-diastolic volume
LVEDVi	left ventricular end-diastolic volume index
LVEF	left ventricular ejection fraction
LVESV	left ventricular end-systolic volume
NLR	neutrophil-to-lymphocyte ratio
NT-proBNP	N-terminal pro-B-type natriuretic peptide
NYHA	New York Heart Association
PARP	poly(ADP-ribose) polymerase
SGLT2	sodium–glucose cotransporter 2
SVI	stroke volume index
T0	baseline time point
