# Supplementary material for: Serum Peroxides and Galectin-3 in Chronic Heart Failure: Cross-Sectional Associations with NT-proBNP and Cardiac Structure
Source: Biomedicines. 2026 Sep 24;14(10):2166. doi: 10.3390/biomedicines14102166 (PMC13701307; doi:10.3390/biomedicines14102166)
Supplement: Supplementary file 1 [file biomedicines-14-02166-s001.zip › Table S1.pdf]

Table S1. Comparison of baseline characteristics between participants included in the complete-case joint biomarker analysis and those with incomplete biomarker data.

Characteristic	Complete-case sample (n = 29)	Incomplete biomarker data (n = 28)	p value
Age, years	71 [67–77]	71 [65–77]	0.9045
Male sex	20/29 (69.0%)	16/28 (57.1%)	0.4173
Body mass index, kg/m^2	29.1 [24.8–31.2]	28.3 [25.9–32.2]	0.9300
NT-proBNP, pg/mL	1101 [820–2690]	1072 [808.8–2148.5][1]	0.8994
LVEF, %	47.6 ± 10.4[2]	41.5 ± 11.6[3]	0.0458
eGFR, mL/min/1.73 m^2	67.9 ± 17.8[4]	62.6 ± 17.4[5]	0.2815
Atrial fibrillation	9/29 (31.0%)	17/28 (60.7%)	0.0343

Data are presented as mean ± standard deviation, median [interquartile range], or n (%), as appropriate. Continuous variables were compared using the Mann–Whitney U test for age, body mass index, and NT-proBNP and Welch's t-test for LVEF and eGFR. Categorical variables were compared using Fisher's exact test. [1] n = 26; [2] n = 28; [3] n = 27; [4] n = 27; [5] n = 25. LVEF, left ventricular ejection fraction; eGFR, estimated glomerular filtration rate; NT-proBNP, N-terminal pro-B-type natriuretic peptide.
